# Influence of Meniscal Repair vs. Meniscectomy on Anterior Cruciate Ligament Reconstruction in Terms of Knee Stability and Radiological Imaging

**DOI:** 10.7759/cureus.43396

**Published:** 2023-08-12

**Authors:** Varun Agarwal, Amit Kale, Ashwinkumar Khandge

**Affiliations:** 1 Orthopedics, Dr. D.Y. Patil Medical College, Hospital and Research Centre, Pune, IND

**Keywords:** knee, osteoarthritis, anterior cruciate ligament reconstruction, meniscectomy, meniscal repair

## Abstract

The menisci are essential components in the pathophysiology of knee osteoarthritis. Patients with meniscal lesions and an intact anterior cruciate ligament (ACL) undergoing meniscectomy exhibit a significantly increased prevalence of radiographic osteoarthritis. ACL reconstruction surgery enhances knee stability and mitigates the advancement of minor meniscal tears. The purpose of this study was to show the influence of meniscal repair as compared to meniscectomy on anterior cruciate ligament reconstruction in terms of knee stability and radiological imaging. This was a retrospective study performed in Dr. D.Y. Patil Hospital, Pimpri, Pune, on a sample size of 30 patients between December 2021 and January 2023. Patients were followed up clinically and radiologically post-operatively at six months and one year. ACL reconstruction with meniscectomy was performed on 16 patients (group 2), while ACL reconstruction with meniscus repair was performed on 14 patients (group 1). At the end of six months in group 1, one patient out of 14 had a grade 2 Lachman test positive, while seven patients out of 16 had a grade 2 Lachman test positive in group 2. It was a statistically insignificant value (p>0.05). Further, at the end of 12 months, X-ray evaluation of the femoral tunnel shows an average increment of 0.5 mm in Group 1, while an average femoral tunnel widening of 3 mm was observed in Group 2. It was statistically insignificant (p>0.05). The study concluded that meniscus repair significantly increases anteromedial knee stability. It has been shown that meniscectomy, when done along with ACL reconstruction, increases the chances of femoral tunnel widening, resulting in less graft bone integration.

## Introduction

The menisci are essential components in the pathophysiology of knee osteoarthritis. Patients with meniscal lesions and an intact anterior cruciate ligament (ACL) undergoing meniscectomy exhibit a significantly increased prevalence of radiographic osteoarthritis, as assessed using the Ahlbäck grading system, with a three- to seven-fold higher incidence. [1] Nevertheless, the establishment of a correlation between radiographic alterations and clinical manifestations remains elusive [2]. Research has demonstrated a notable increase in the occurrence of osteoarthritis, ranging from three to seven times higher, in individuals who have undergone either a partial or complete meniscectomy. ACL reconstruction is widely recognized as the prevailing treatment method for restoring knee stability, facilitating a quicker return to sports activities, and reducing the time required to resume regular daily activities, even in cases where meniscal injuries are present [3].

ACL reconstruction surgery enhances knee stability and mitigates the advancement of minor meniscal tears. Menisci that cannot be repaired are managed through excision and stabilization of the rim, whereas those that can be repaired are treated using meniscus repair techniques [4].

The consequences of the early rebuilding of the ACL combined with meniscal repair were found to be superior to those of delayed repairs, exhibiting higher rates of successful healing. The procedure of meniscectomy has been demonstrated to have adverse consequences for the knee, specifically leading to heightened contact pressure in the tibial and femoral condyles, early manifestations of osteoarthritis, and rotatory instability [4].

The selection between meniscectomy and meniscus repair during ACL reconstruction is contingent upon the condition of meniscal tears and their potential for successful repair at the moment of surgical intervention. Numerous studies have provided evidence indicating superior outcomes associated with partial meniscectomy as compared to total meniscectomy in terms of weight transmission [5], anterior-posterior stabilization of the joint [6], and proprioception [7-9]. However, there isn't enough information in the literature currently available to establish how meniscus repair affects knee stability after anterior cruciate ligament (ACL) restoration. Our goal is to find out if meniscus repair during ACL reconstruction may affect the knee's anteromedial stability.

## Materials and methods

This retrospective study was conducted at Dr. DY Patil Hospital, Pune, between December 2021 and January 2023. ACL restoration employing hamstring grafts was conducted on the patients. Meniscus repair, or meniscectomy, was done during the corresponding procedure. Patients were followed up postoperatively at six months and one year, both clinically and radiologically. Knee X-rays were done postoperatively and at the end of one year. Clinically, patients were evaluated for anteromedial stability by the Lachman test.

Inclusion criteria

Patients with the following conditions were included in the study: A) A complete ACL tear with a medial or lateral meniscus tear is eligible for the all-inside technique. B) Young, physically active male or female age group 18 to 30 years. C) Patient willing to give consent for the study. D) Patient willing to undergo rehabilitation after surgery. 

Exclusion criteria

Patients with the following conditions were excluded from the study: A) Medial compartment osteoarthritis (both clinically and radiologically). B) Associated multi-ligament injury. C) Associated chondral injury. D) Revision ACL reconstruction.

After admission and confirmation of the injuries on the MRI knee, patients were counseled, consented, and operated on for anatomical ACL reconstruction using a hamstring graft arthroscopically. The graft was secured on the femoral side utilizing the Smith and Nephew Ultra button, while on the tibial side, it was affixed using the Smith and Nephew BIOSURE. The intricacy of the rip, its position inside the meniscus, and how long it has been since the incident were among the considerations that went into the decision about the meniscal injury. For meniscus rips that are considered to be repairable and lie within the red-red and red-white zones, we offer surgical intervention. Additionally, we limit our treatment to cases where the duration of the injury is less than six months. The tears, which were complex, older than six months, and in the white-white zone, were treated with meniscectomy with peripheral rim stabilization. Post-operative X-rays were done to calculate the femoral tunnel diameter.

Patients were monitored at six months and one year in the outpatient department, where the Lachman test was used to assess anteromedial knee instability. Patients with more than Grade 1 instability on the Lachman test were considered unstable. Follow-up X-rays were done at the end of one year, and femoral tunnel diameter was evaluated.

Statistical analysis

Data was collected in MS Excel 365 (Redmond, USA) and analyzed in IBM Corp. Released 2022. IBM SPSS Statistics for Windows, Version 29.0. Armonk, NY: IBM Corp. Frequency analysis was performed on qualitative data, and descriptive analysis was performed on quantitative data. The difference between the two groups was determined through an independent t-test. The p-value was significant at a 95% confidence interval.

## Results

**Figure 1:**
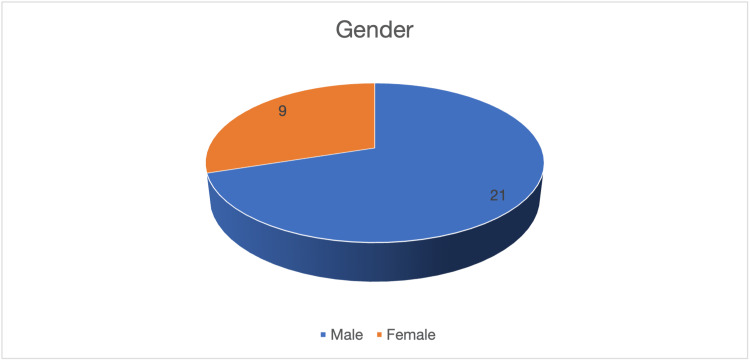
Gender distribution

We followed a total of 30 patients with ACL and meniscus injuries. Patients were in the age group of 18-30 years, with an average age of 24 (SD = 4.65) years. All patients have traumatic knee injuries. There were nine females and twenty-one males (Figure 1).

Within this cohort, a total of 20 patients exhibited a medial meniscus tear, while eight patients presented with a lateral meniscus tears. Additionally, two patients were diagnosed with both medial and lateral meniscus tear. Out of a total of 30 cases of meniscus tears, 14 were deemed suitable for repair, while the remaining 16 patients necessitated meniscectomy due to the tear's complexity and duration. The average time from injury to surgery for the whole sample was 3.5 (SD = .04 weeks). The average time from injury to surgery for repairable meniscus and non-repairable meniscus was 3.3 (SD = .15 weeks) and 3.7 (SD = .43) weeks, respectively. The surgical procedure employed for meniscus repair involved the utilization of the all-inside technique, specifically the Smith and Nephew fast fix system. The average hamstring graft diameter was 8.5 (SD = 0.94) mm with a minimum graft diameter of 8 mm and a maximum of 9 mm on the femoral side, and 8.5 (SD = 1.01) mm with a minimum graft diameter of 8 mm and maximum of 9.5 mm on the tibial side. The average femoral tunnel size intra-operatively was 8.5 (SD = 0.98) mm.

**Figure 2:**
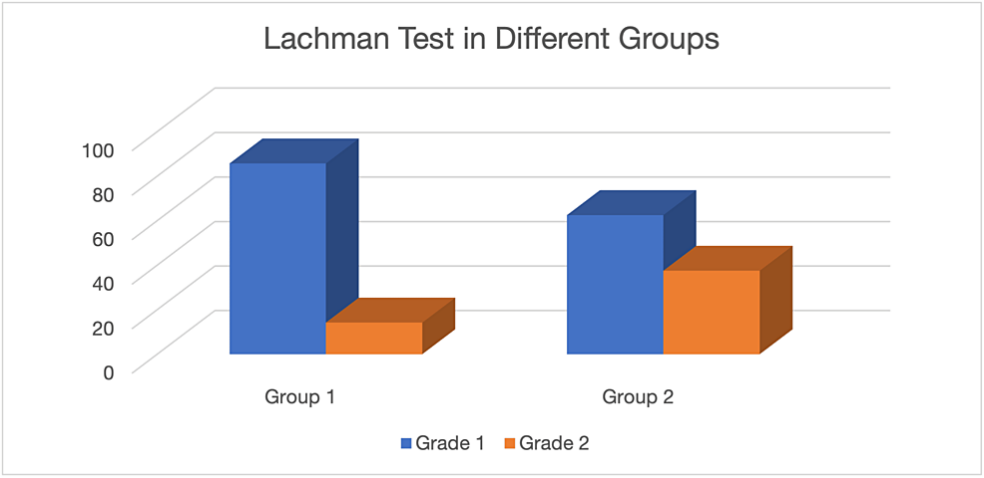
Lachman test in different groups

ACL reconstruction with meniscectomy was performed on 16 patients (group 2), while ACL reconstruction with meniscus repair was performed on 14 patients (group 1). At the end of six months in group 1, one patient out of 14 had a grade 2 Lachman test positive, while seven patients out of 16 had a grade 2 Lachman test positive in group 2. It was a statistically insignificant value (p>0.05) (Figure 2).  

**Figure 3:**
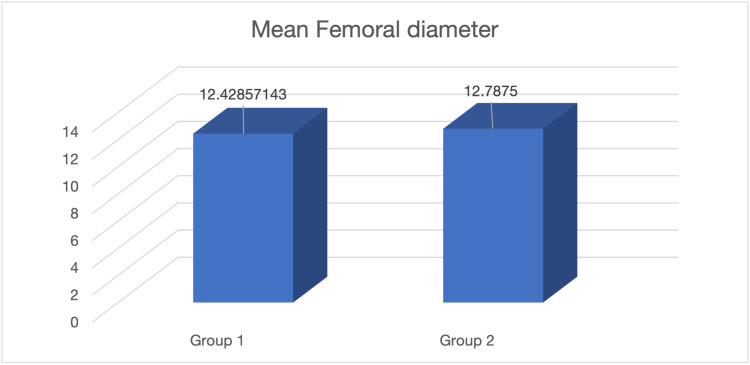
Mean femoral diameter

At the end of 12 months (Table 1), X-ray evaluation of the femoral tunnel shows an average increment of 0.5 mm in Group 1, while an average femoral tunnel widening of 3 mm was observed in Group 2. It was statistically insignificant (p>0.05) (Figure 3).

**Table 1:** Femoral tunnel diameter in different groups

Group	No. of patients	Pre-op mean in mm	Femoral diameter at end of 12 months mean in mm	P-value
Group 1 (Meniscus repair)	14	8.5	12.42±1.03	.432
Group 2 (Meniscectomy)	16	8.5	12.78±1.36	

## Discussion

The meniscus is an important and essential structure that acts as a shock absorber in the knee. It has been proven beyond doubt that meniscectomy increases cartilage stresses, leading to early osteoarthritic changes in injured knees. There is a 77% risk of meniscus injury along with an ACL injury. Both arthroscopic ACL reconstruction with meniscus repair and arthroscopic ACL reconstruction with meniscectomy are often used to treat ACLs with meniscus injuries. The role of meniscus as a stabilizer has not been well established [10].

The ACL stabilizes the knee both in the coronal plane and against rotational pressure. In the knee joint, the menisci’s role is important. Along with cruciate ligaments, particularly rotary stability, menisci provide knee stability in addition to basic functions, including shock absorption, enhancing joint congruity, and distributing body weight [10].

According to studies, up to 87% of patients with ACL injuries treated conservatively had fair to poor outcomes. As little as 6% of patients return to employment or sports after rehabilitation. A decreased performance level was also seen in 33% of former athletes [11]. ACL restoration has withstood the test of time to restore knee stability with a quicker return and shorter recovery time. A lot of patients choose conservative measures like bracing and physiotherapy. They run a greater chance of twisting knees again and injuring the meniscus if they try to resume high-intensity activities. Around 50% to 68% of traumatic ACL tears also include meniscal tears, according to research [12]. Together, they have the potential to seriously impair both athletes and non-athletes. 

Meniscus repair has received a lot of attention recently because of improved anatomical understanding, improved fixation techniques, and improved implant technology. Still, meniscus shaving may be the preferred course of action in some cases with irreversible rips. Indications of meniscus repair include: 1) any peripheral non-degenerative longitudinal tear < 3 cm, 2) if the tear is within 3 mm of the periphery, it is considered vascular, 3) unstable or vascular tears > 7 mm are repairable, and 4) mobile, single, vertical, longitudinal tear of the meniscus limited to the vascular outer one-third of the meniscal substance. Excision and rim stabilization are used to treat menisci that cannot be repaired. Those that can be repaired are handled with meniscus repair using various methods. Even though untreated meniscal injuries following ACL repair do not have poor short-term functional scores for up to three years, the long-term prognosis is not favorable [13]. They are more likely to develop osteoarthritis. In their 12-year follow-up study of young female football players, Lohmander et al. reported that untreated ACL injuries with clinical laxity had a very high frequency of radiographic osteoarthritis, functional impairments, and pain [14]. 

ACL reconstruction has been shown to significantly lower the risk of OA. Additionally, the development of OA [15] appears to be significantly impacted by early injuries linked to ACL tears, such as meniscal tears and chondral lesions. It has been demonstrated that as time passes following ACL damage, meniscal and chondral problems become more common. According to Granan et al., after an ACL injury, the chance of cartilage damage increases by 1% per month. On ACL early reconstruction, there is agreement. Hur et al. have demonstrated that early ACL reconstruction increased the likelihood of early meniscal repair with favorable healing rates compared to late repairs. When treatment was finally provided after three months, meniscal injuries increased to 70% and were only 17% more likely to be repaired. Additionally, meniscal tears grew distorted or advanced to complete tears and developed loose bodies when patients arrived late for therapy. Those tears needed to be dried up. Additionally, there is proof to support the idea that an early ACL restoration can help patients restore good knee motion, postural control, and stability [16].

According to Dilworth et al., as compared to isolated meniscal tears, ruptured ACLs with meniscal tears have greater healing capacity [16]. The arguments put forth are as follows: 1) An ACL reconstruction improves the biomechanical stability of the knee, preventing repeated meniscus injuries 2) Compared to isolated meniscal tears, fibrin clots present in ACL tears create a more favorable environment for meniscal repair. Fibrin clot, which is similar to PRP in terms of the components and healing mechanism, can be prepared with less effort and in a shorter time without expensive equipment and causes few side effects with the use of autologous blood. The fibrin clot acts as a hematoma that promotes healing of the meniscus. In other words, lesions that had been filled with a fibrin clot healed through a proliferation of fibrous connective tissue that modulated into fibrocartilaginous tissue.

As joint filers, menisci improve the congruence of the femoro-tibial surface, sharing approx. 50% of the body weight. They also serve various auxiliary purposes, such as feeding the cartilage with synovial fluid and preventing capsular impingement during knee motion. Along with the cruciate ligaments, they also help with mechanical knee stability, particularly rotary stability, which adds an extra gliding function to the hinge-type knee joint [10].

Meniscectomy negatively affects anterior stability, load distribution, and joint alignment [17-19]. In comparison to lateral meniscectomy, which raises contact stresses by 200% to 300%, tibio-femoral contact stresses increase by 100% due to medial meniscectomy. Since the hoof tensions across the meniscal fibers are eliminated, a single radial cut is comparable to a meniscectomy. According to Lorbach et al., medial meniscectomy causes the anterior tibial translation in ACL-deficient knees to rise [18]. Due to its physical proximity to the popliteus tendon and the posterolateral capsule, total lateral meniscectomy is also linked to an increase in postoperative posterolateral rotational instability.

In knees with ACL reconstruction, Laprade et al. have demonstrated the significance of the lateral meniscus. When the posterior lateral meniscal root is disrupted following ACL reconstruction, they discover greater anterior translation and rotatory instability of the knees [20]. This could put further strain on the ACL transplant. Meniscectomy following ACL reconstruction and tunnel widening, however, have never been linked. Harner et al. divided meniscal tears into three categories based on their location: white zone, red-white zone, and red zone. This helps to forecast the tear's likelihood of healing after repair [10]. Greater healing potential exists for tears in peripheral vascular zones.

By using a second-look arthroscopy, Matsushita et al. [21] found that 56% of patients had fully healed meniscus tears. Yang et al. have described the refreshing of the RAMP lesion following ACL reconstruction, and all inside approaches have demonstrated equivalent results. This might be because these peripheral tears have a better chance of healing because they are in a circulatory zone. Meniscus preservation has received much attention recently and is preferred over meniscectomy operations.

These tears are, nonetheless, sometimes irreversible, distorted, and avascular and can harm intact articular cartilage when patients arrive late. The surgeon's reasonable alternative in these circumstances is a partial or subtotal meniscectomy. Subtotal meniscectomy does not recover if it is in an avascular zone, but total meniscectomy exposes the vascular peripheral bed for regeneration.

ACL reconstruction surgery complications include bony tunnel expansion. A significant association between tibial tunnel expansion and anterior knee laxity was discovered by Webster et al. Bone tunnel widening has a number of causes, including the kind of graft, the elastic or rigid graft fixation technique, the single or double bundle fixation, and vigorous rehabilitation [22]. According to the biological idea, osteolysis can be brought on by the production of nitric oxide, interleukins, and TNF from the synovial fluid. Graft tunnel micromotion in suspensory fixation, stress shielding near the interference screw, and non-antomical graft fixation are the mechanical causes of tunnel widening [22-24]. 

In our investigation, we discovered that, despite being statistically insignificant (p = 0.432), the amount of tibial tunnel widening was less in the meniscus repair group than in the meniscectomy group, measuring 12.42 mm (S.D. = 1.03) compared to 12.27 mm (S.D. = 1.36). In comparison to 7.14% of the meniscus repair group, 43.75% of the meniscectomy group scored grade 2 on the Lachman's Test (p>.05). Even if the functional scores don't change substantially, long-term follow-up may show a considerable difference.

At the end of the year, the femoral tunnel had widened in the meniscectomy group compared to the meniscus repair group. Tunnel widening after ACL reconstruction is caused by various mechanical and biological factors. The difference in the graft fixation device is one such factor. According to some writers, cortical button attachment promotes what are known as the bungee effect and wind-shield wiper effects, respectively, of the graft at the intra-articular aperture and inside the bone tunnel [25]. The shorter tunnels may lessen these effects if the cortical fixation device is placed closer to the joint space [26]. Tunnel positioning is another potential influence. The graft-bone contact may be subjected to more stress if the graft-bending angle of an ACL reconstruction is more acute at the tunnel's aperture. Bioabsorbable graft fixation material and synovial fluid extravasations are biological factors in tunnel widening. A chemical osmotic effect within the bone tunnel may cause the disintegration of bioabsorbable screws to encourage tunnel enlargement. This signifies less stability in the meniscectomy group. It may have a detrimental effect on graft-bone integration, which was not studied in this series, but it has been documented in the literature that femoral tunnel widening is associated with ACL failure.

Limitations of the study

The main limitation of the study was its small sample size. Due to limited time, we could not include a higher number of participants, so there are chances that it may affect the overall reliability of the study. Another limitation of this study was its brief follow-up time.

## Conclusions

In our series, it has been observed that meniscus repair significantly increases anteromedial knee stability. It has been shown that meniscectomy, when done along with ACL reconstruction, increases the chances of femoral tunnel widening, resulting in less graft bone integration. Understanding the function of meniscus healing in ACL integration after ACL restoration requires further research.
